# Rare case of symmetrical peripheral gangrene due to septic shock, disseminated intravascular coagulation and inotropic use

**DOI:** 10.1016/j.amsu.2018.09.025

**Published:** 2018-09-25

**Authors:** Miguel N. Albano, Sofia G. Brazão, Teresa V. Caroço, João M. Louro, Maria I. Coelho, Carlos E. Costa Almeida, Luís S. Reis, Carlos M. Costa Almeida

**Affiliations:** Centro Hospitalar e Universitário de Coimbra (Hospital Geral - Covões), S. Martinho de Bispo, 3041-853, Coimbra, Portugal

**Keywords:** Case report, Symmetrical peripheral gangrene, Septic shock, Disseminated intravascular coagulation, Inotropic use

## Abstract

**Introduction:**

Symmetrical peripheral gangrene (SPG) is a rare syndrome defined by the peripheral ischemic lesion of two or more extremities in the absence of major vascular obstructive disease.

**Presentation of case:**

A 45yo woman, admitted in intensive care unit due to urinary septic shock, in need of high doses of amines, developed cold extremities with acrocyanosis that rapidly progressed to gangrene. Laboratory analysis revealed increased inflammatory parameters, liver shock, thrombocytopenia, prolonged coagulation times, increased D-Dimers and isolation of *Acinetobacter baumanni* in urine culture. An intravenous vasodilator was initiated with clinical benefits. After improvement and delimitation of the lesions, the patient underwent the amputation of the distal phalanges of the 2nd, 3rd and 4th fingers of the right hand and the toes of both feet.

**Discussion/conclusion:**

Even though there is no consensus regarding SPG treatment, consequences should be mitigated, particularly when vasodilators are used, in order to avoid major amputation.

## Highlights

• Symmetrical Peripheral Gangrene (SPG), although rare, must be immediately recognized because of its harmful consequences.
• SPG has a high mortality rate and a high percentage of the survivors have at least one limb amputated.
• The necessary measures should be taken in a timely manner to mitigate consequences.
• Treatment of the infection, the avoidance of vasopressors, and the use of LMWH and vasodilators, seem to be important to reduce major amputation.

## 1 Introduction

Symmetrical peripheral gangrene (SPG) is a rare syndrome defined by a peripheral ischemic lesion of two or more extremities in the absence of major vascular obstructive disease. It was described for the first time in 1891 by Hutchinson in a clinical case of sepsis with intravascular disseminated coagulation [1].

The differential diagnosis include frostbite, ergotism, vasospasm (idiopathic or scleroderma-associated Raynaud's phenomenon), calciphylaxis, postoperative thrombotic thrombocytopenic purpura, myeloproliferative or lymphoproliferative disorders (including monoclonal gammopathies), vasculitis, rheumatologic or immunologic disorders and the antiphospholipid syndrome and uncontrolled proinflammatory disorders such as ulcerative colitis [2].

This is a case of symmetrical peripheral gangrene due to septic shock secondary to urinary tract infection with intravascular disseminated coagulation and the use of high doses of inotropics, which is a rare syndrome with harmful consequences frequently ending up in major amputation of several limbs.

Given the high mortality and morbidity rates, the diagnosis should be fast, and correct treatment instituted. However, published cases show different approaches and outcomes.

This work has been reported in line with the SCARE criteria [3].

## 2 Presentation of case

A 45yo woman presented at the emergency department with low back pain and anuria in the previous 24 hours. Laboratory investigations indicated leukocytosis (46700 × 10ˆ9/L), CRP of 16,9 mg/dL and acute renal failure (creatinine of 2.57 mg/dL). Arterial blood gases revealed metabolic acidosis (pH 7.21), increased lactates (5.58 mmol/L) and low bicarbonate (12.1 mmol/L).

Clinical aggravation occurred with a septic shock, requiring intubation, mechanical ventilation and the use of high doses of vasopressors (norepinephrine > 2 mcg/kg/min and dopamine > 10 mcg/kg/min).

The patient was admitted to intensive care unit with the diagnosis of septic shock secondary to urinary tract infection.

**Fig. 1:**
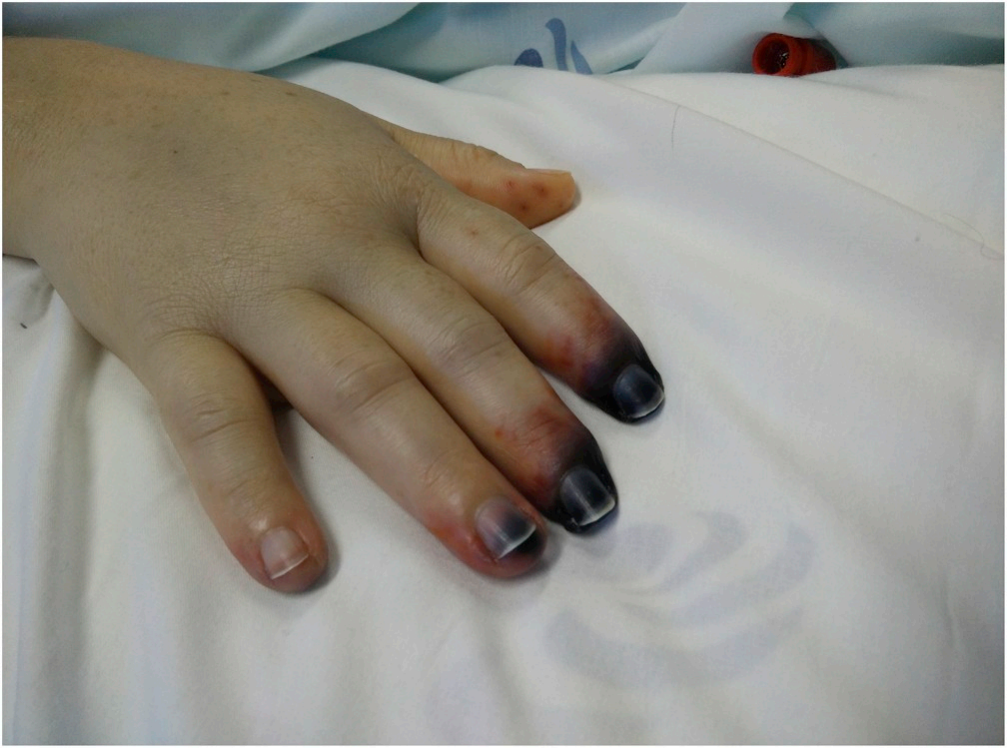
Right hand with acrocyanosis.

**Fig. 2:**
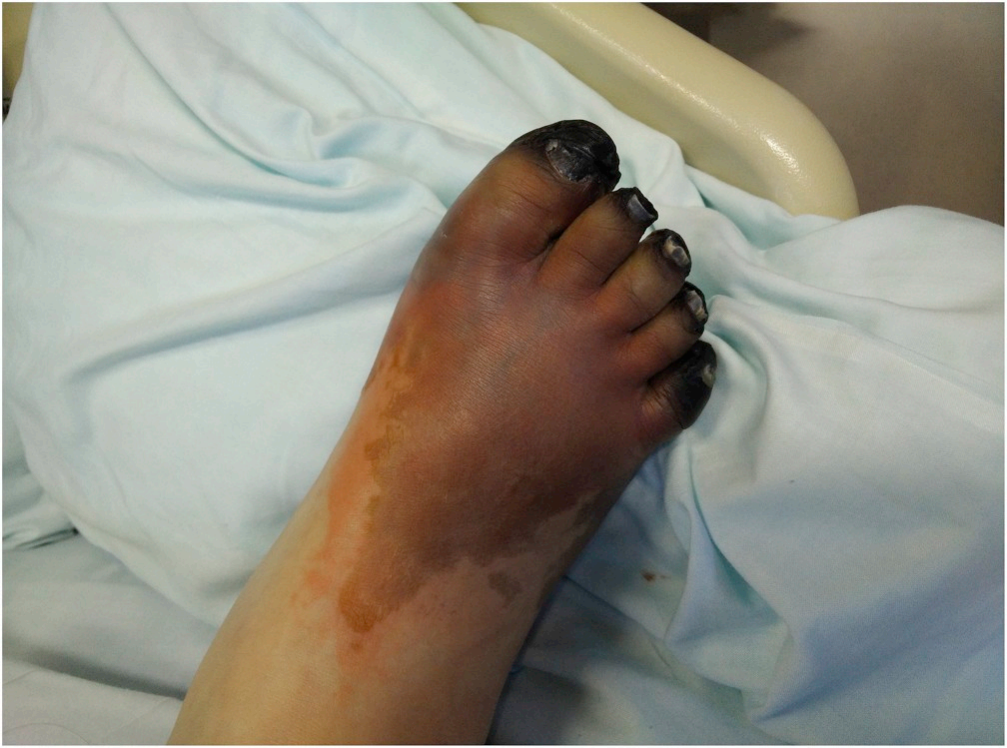
Right foot with acrocyanosis and peripheral necrosis.

**Fig. 3:**
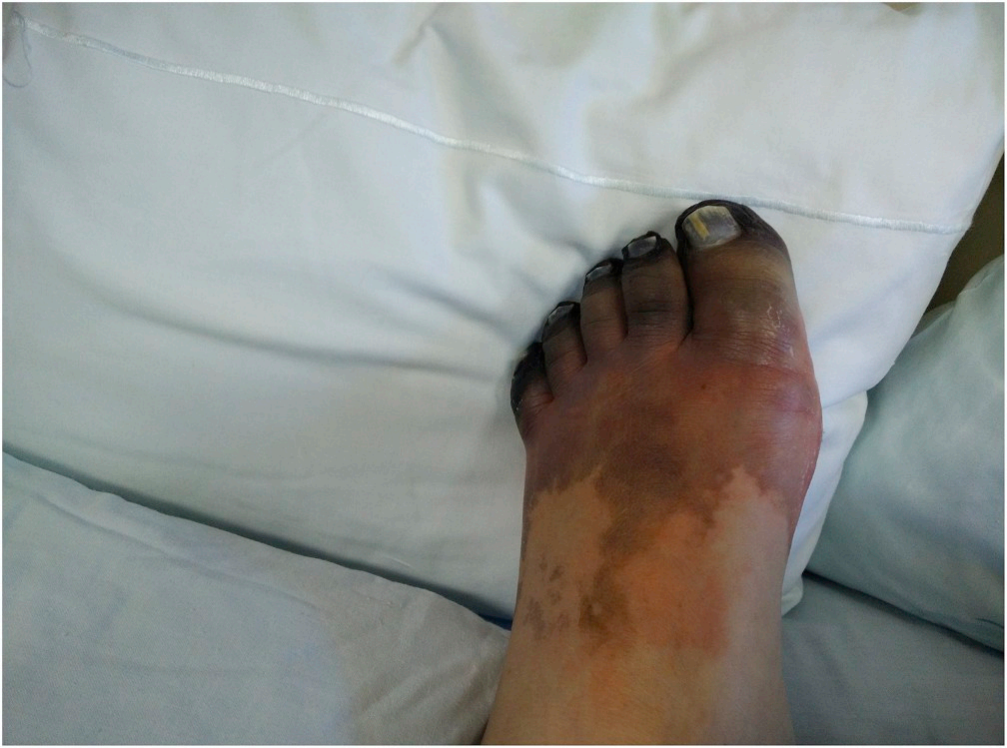
Left foot with acrocyanosis and peripheral necrosis.

Five days after admission, the patient developed cold extremities with acrocyanosis of the right hand and both feet, although with audible pulse. The lesions rapidly evolved to gangrene (Fig. 1, Fig. 2, Fig. 3). Laboratory investigations showed increased inflammatory parameters, acute renal injury, thrombocytopenia (29 × 10ˆ9/L), and prolongation of coagulation times (Prothrombin time: 22.2s and INR of 2.03) with increased D-Dimers (11710 ng/mL) and low fibrinogen of 96mg/dL, revealing intravascular disseminated coagulation. Microbiological exams showed positive urine culture for *Acinetobacter baumanni*. At this time, the patient no longer required mechanical ventilation or vasopressors and was being treated with large spectrum antibiotics, LMWH and correction of hydro-electrolytic disturbances. Isosorbide dinitrate was started but without any clinical improvement and substituted for prostaglandin I2 analogue at day 13. Amelioration of the cyanotic regions was observed.

**Fig. 4:**
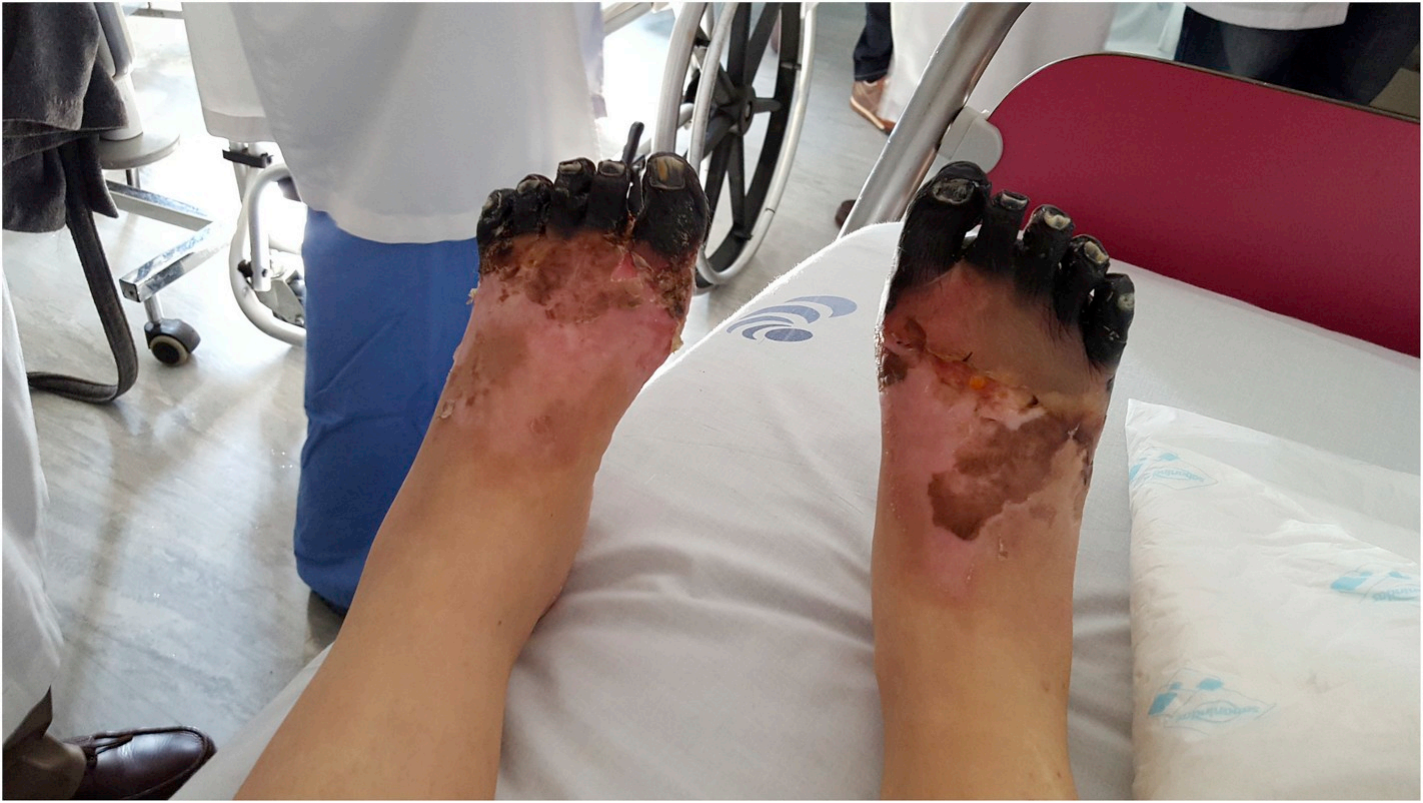
Delimitation of the necrosis of both feet.

**Fig. 5:**
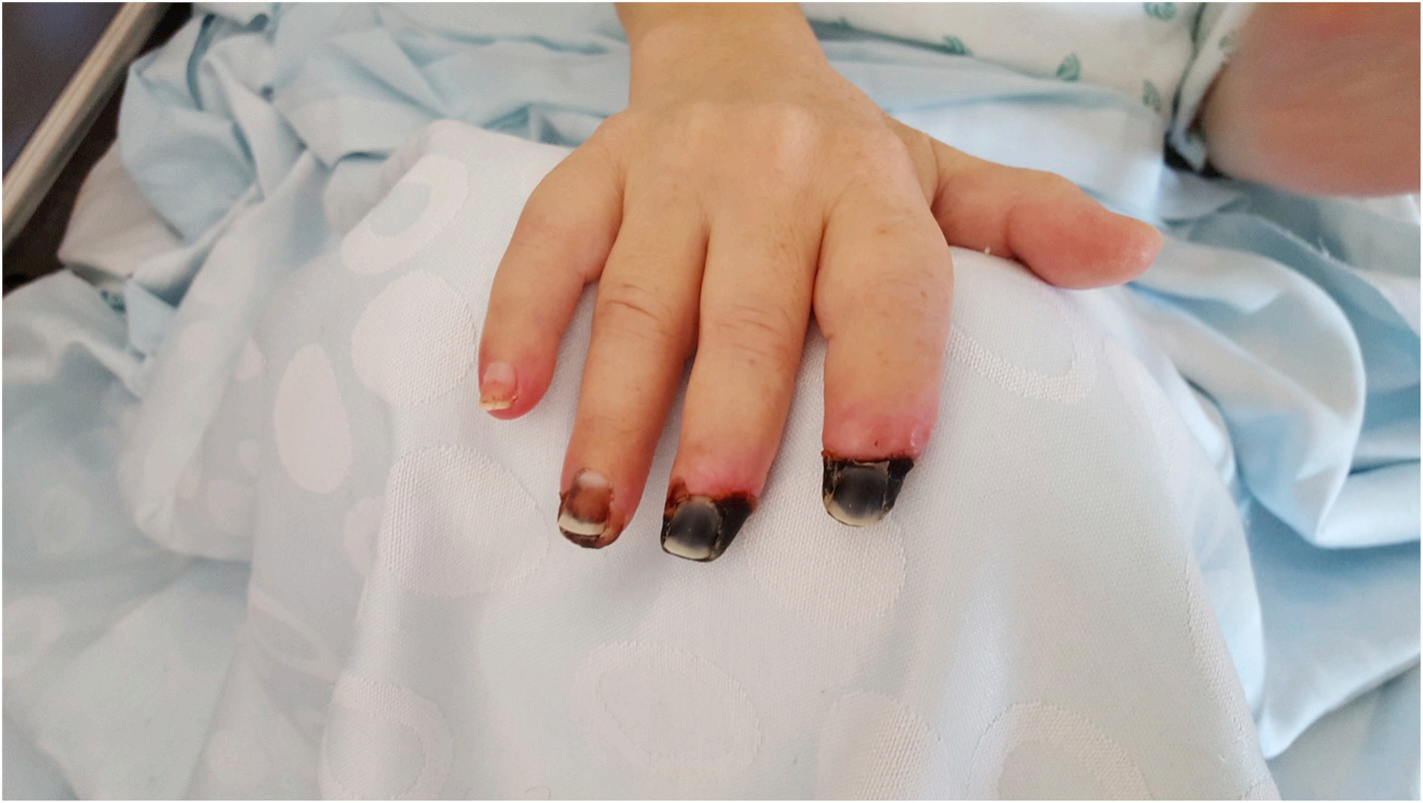
Delimitation of the necrosis of right hand fingers.

**Fig. 6:**
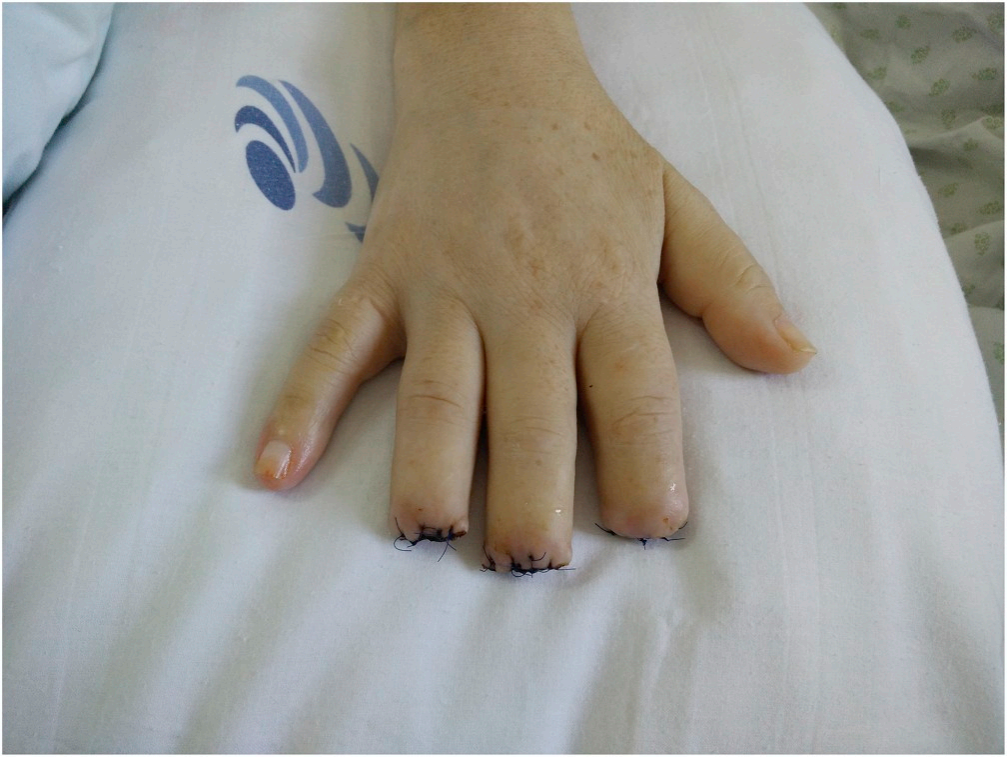
Right hand after amputation of the distal phalanges.

**Fig. 7:**
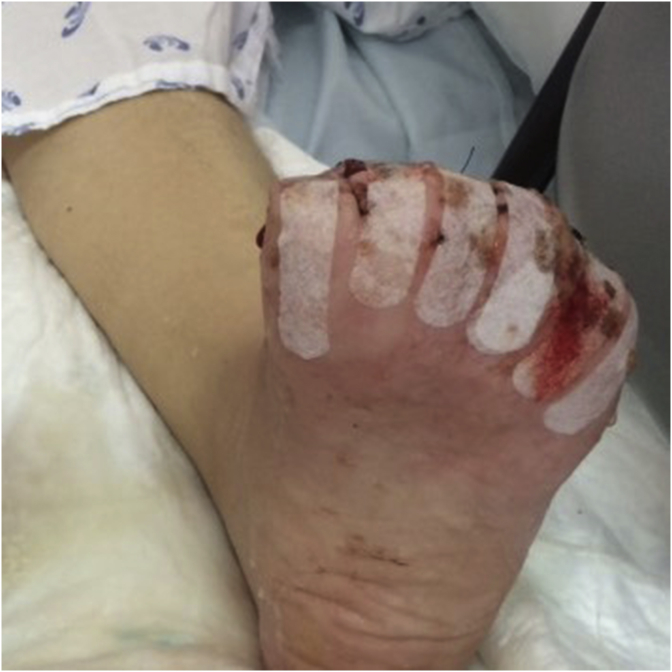
Left foot after toe amputation.

At day 21, the patient was transferred to the Surgery ward, where, after the improvement of both the initial lesions and the delimitation of lesions (Fig. 4, Fig. 5), she underwent the amputation of the distal phalanges of the 2nd, 3rd and 4th fingers of the right hand and the toes of both feet (Fig. 6, Fig. 7). Wound care with paraffin gauze dressings and Cronocol^®^ (sterile, biodegradable implant containing gentamicin sulfate) was provided.

Given the progress of the surgical wounds, the patient was discharged home with the above-mentioned wound care dressings.

**Fig. 8:**
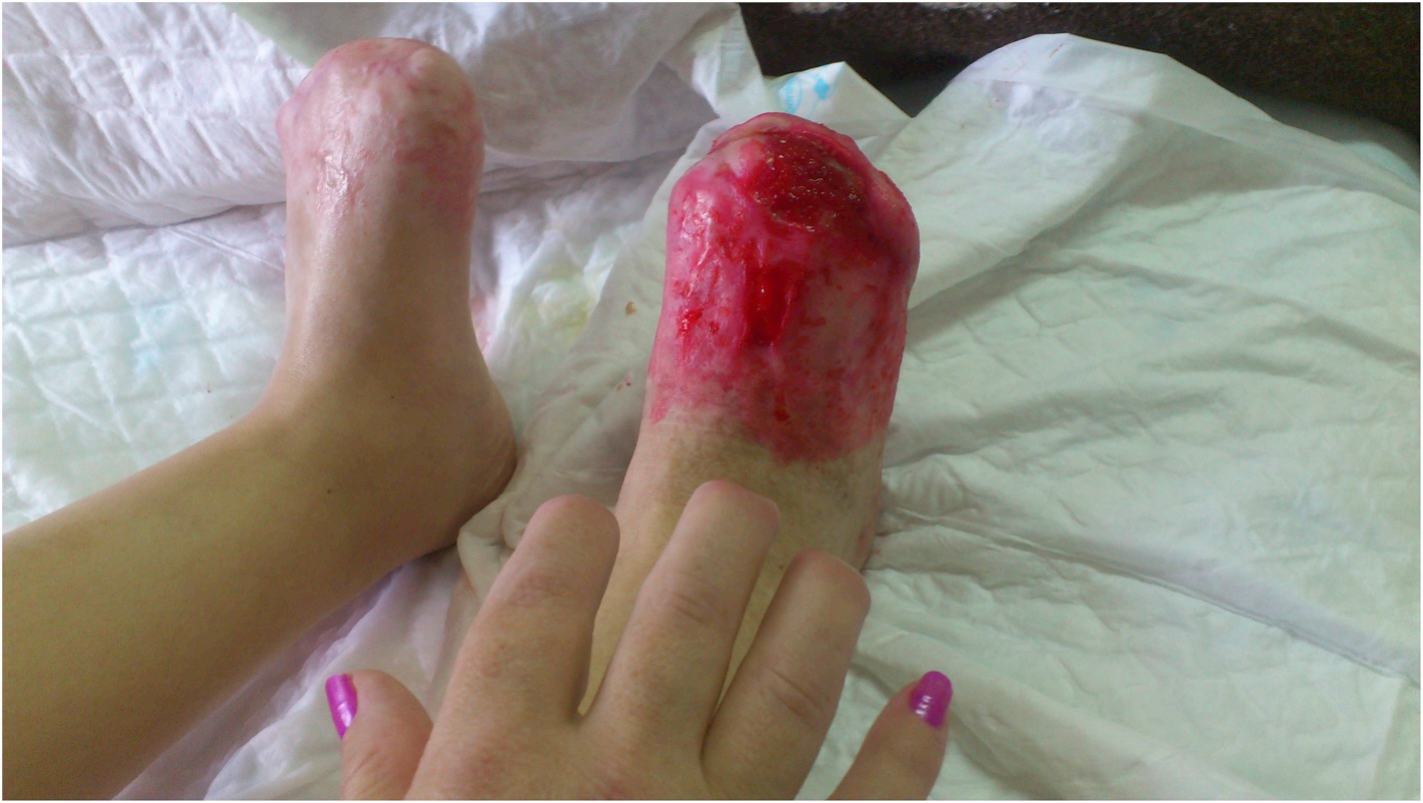
Complete healing of the operative wounds after 2 month.

At two months follow-up, the patient presented completely healed operative wounds (Fig. 8).

## 3 Discussion/conclusion

Symmetrical peripheral gangrene (SPG) is a rare syndrome defined by a peripheral ischemic lesion of two or more extremities in the absence of major vascular obstructive disease. It was described for the first time in 1891 by Hutchinson in a clinical case of sepsis with intravascular disseminated coagulation [1].

Clinically, it may be presented with signs of ischemic limb injury, sharply demarcated and strikingly symmetric that progresses rapidly to necrosis with palpable pulses [3].

Typically associated with thrombocytopenia and coagulopathy in critically ill patients. Metabolic acidosis and acute ischemic hepatitis may be present, with the onset of ischemic limb necrosis beginning two to five days after the initial elevation in liver enzymes [3].

The pathogenesis of SPG is not well known. However, it is thought to be related to any condition which decreases the blood supply, and consequently the delivery of nutrients and oxygen, to the more peripheral regions for an extended time [4]. Microcirculatory alterations, vasospastic conditions, such as the use of vasoactive drugs, and states of low cardiac output are considered as the most frequent associations with SPG. That's why the differential diagnosis could be wide and dependent on the clinical context [5].

Disseminated intravascular coagulation (DIC), often as a consequence of infection, appears to be frequently associated with SPG. Literature reports its occurrence in about 85% of cases [4]. The most frequently implicated microorganisms are pneumococcus, streptococcus and staphylococcus. However, cases of bacterial gram-negative infections, viral infections and infections by protozoa such as Plasmodium falciparum have also been described [5,6].

We describe a case of SPG in a patient with septic shock and DIC secondary to urinary tract infection by *Acinetobacter baumanii* and the use of high doses of noradrenaline and dopamine. The hypotension and vasopressor therapy reduce the blood flow into the distal extremities, and therefore predispose the patient with a septic shock with DIC to microthrombosis and a consequent ischemia and progression to necrosis and gangrene. In a brief review of literature, we found no cases of SPG with DIC associated with an *Acinetobacter baumanii* infection. We also observed that the majority of the cases reported major amputations up to the knees and elbows [2,4,7,8].

SPG associated with DIC has a mortality rate of 35%; nevertheless, about 50% of survivors require amputation of at least one limb [6].

This entity does not have a therapy based on controlled clinical trials, or even a well-defined therapy, perhaps because of its rarity. However, if a precipitating factor is identified, it seems indisputable that it must be treated or, in the case of a drug, suspended. Several therapeutic attempts were found in literature: epoprostenol (prostaglandin) plus tissue plasminogen activator [9]; epoprostenol plus plasmaferesis [10]; combination of plasmapheresis, leukapheresis and antibiotic [11]; sympathetic blockade [12]; nitroglycerin ointment [13]; aspirin [14]; heparin [6]. However, it should be noted that rarely do medical therapeutic attitudes seem to prevent the progression of the disease or reverse the already gangrenous areas [4]. After the patient's general condition is improved and the lesions are demarcated, the patient is submitted to surgery, which consists of the amputation of the irreversible areas.

In our case, the combined medical treatment (large spectrum antibiotics, low molecular weight heparin (LMWH) and prostaglandin I2 analogue) was used, which allowed both the improvement of lesions and the delimitation of irreversible lesions, thus providing the possibility of minor mutilating amputation.

## Consent

Written informed consent was obtained from the patient for publication of this case report. A copy of the written consent is available for review of this journal on request.

## Provenance and peer review

Not commissioned, externally peer reviewed.

## Ethical approval

This paper is exempt of ethical approval from my institution.

## Sources of funding

This research did not receive any specific grant from funding agencies in the public, commercial, or not-profit sectors.

## Author contribution

Miguel N. Albano – study concept and design, data collection and analysis, writing the paper, review.

Teresa V. Caroço – Review.

Sofia G. Brazão - Review.

João M. Louro – Review.

Maria I. Coelho – Study concept and design, data collection and analysis, review.

Carlos E. Costa Almeida– Review.

Luis S. Reis – Review.

Carlos M. Costa Almeida – Review.

## Conflicts of interest

Authors declare no conflicts of interest.

## Research registration number

researchregistry2404.

## Guarantor

Luís S. Reis; Carlos M. Costa Almeida.

## Appendix A Supplementary data

SCARE Guideline Checklist SCARE Guideline Checklist
